# Evidence-based nursing strategies for the prevention and management of oral mucositis in hematopoietic stem cell transplantation patients

**DOI:** 10.3389/fmed.2026.1737301

**Published:** 2026-01-26

**Authors:** Min Cui

**Affiliations:** Department of Hematology, The First Affiliated Hospital of Harbin Medical University, Harbin, China

**Keywords:** clinical pathway, evidence-based nursing, hematopoietic stem cell transplantation, management strategy, multidisciplinary collaboration, oral mucositis, patient-centered care, supportive care

## Abstract

Hematopoietic stem cell transplantation (HSCT) is a cornerstone therapy for hematological malignancies, frequently complicated by treatment-related oral mucositis (OM). This complication leads to severe pain, nutritional compromise, heightened infection risk, and may result in treatment delays, prolonged hospitalization, and diminished long-term health-related quality of life. While clinical guidelines exist, a significant gap persists between evidence and practice, especially in the systematic and individualized application of preventive strategies. From an evidence-based nursing perspective, this perspective article proposes a comprehensive management framework. This framework integrates evidence synthesis, patient-specific risk assessment, dynamic monitoring, and multidisciplinary collaboration to align standardized interventions with personalized patient needs across all phases of HSCT. By synthesizing current evidence, analyzing practical challenges, and proposing a structured management pathway, this perspective article aims to guide clinical practice, inform future research, and ultimately improve care standards for OM in HSCT patients.

## 1 Introduction

### 1.1 Severity of the problem

Hematopoietic stem cell transplantation (HSCT) is a critical and potentially curative intervention for malignant hematological disorders, certain solid tumors, and genetic immunodeficiencies (1, 2). However, the necessary pre-transplant conditioning regimens—involving high-dose chemotherapy and/or radiotherapy—directly damage the rapidly dividing basal epithelial cells of the oral mucosa (3). Consequently, oral mucositis (OM) is an exceedingly common complication, with a reported incidence of 70 to 100% following HSCT (3, 4). Approximately one-third to one-half of these cases are severe [World Health Organization (WHO) Grades III-IV], representing a major clinical management challenge (5).

The pathogenesis of HSCT-associated OM is now recognized not as a simple inflammatory response, but as a dynamic and complex biological process (6). It is initiated by direct cytotoxic injury, which triggers a cascade of molecular and cellular events (7). The upregulation and release of pro-inflammatory cytokines (e.g., tumor necrosis factor-alpha, interleukins-1β and interleukins-6) amplify tissue damage and mediate significant pain (6, 8). Subsequent loss of mucosal barrier integrity permits the translocation of oral commensal microorganisms into the bloodstream, substantially increasing the risk of life-threatening systemic infections (7, 8). Therefore, OM is best characterized as a multifaceted biological injury, driven by a dysregulated cytokine network and shaped by interactions between the epithelium, submucosa, and local microbiome (6, 9).

The risk and severity of OM differ substantially between autologous and allogeneic HSCT. Autologous transplantation primarily involves acute, chemotherapy-induced mucosal toxicity, which is often self-limiting (10). In contrast, allogeneic HSCT entails more intensive conditioning, prolonged immune dysregulation, immunosuppressive therapy, and the potential for graft-versus-host disease, all of which contribute to more severe, persistent, and complex mucosal damage (11). This distinction necessitates differentiated nursing assessment strategies, with allogeneic recipients requiring closer monitoring for prolonged ulceration, secondary infection, and chronic oral sequelae (12).

The impact of OM on patients is profound and multidimensional. Acutely, severe pain frequently impedes oral intake, leading to malnutrition, dehydration, and a common reliance on opioid analgesia and total parenteral nutrition (13). The ulcerated mucosa provides a portal for pathogenic entry, increasing the risk of bacteremia and sepsis, which may increase transplant-related mortality (14, 15). Furthermore, severe OM can delay hematopoietic engraftment, prolong hospitalization, increase treatment costs, and potentially compromise therapeutic efficacy by necessitating treatment modifications (5, 14). Beyond the physical sequelae, the associated pain and functional impairment contribute to significant psychological distress, including anxiety and depression, adversely affecting health-related quality of life (HR-QOL) long after clinical recovery (16, 17).

### 1.2 Rationale for evidence-based nursing

The complexity of OM makes reliance on anecdotal experience or routine care insufficient for optimal management. Consequently, integrating an evidence-based nursing (EBN) framework is essential. EBN is a systematic, problem-solving approach to clinical decision-making that combines the best available research evidence, clinician expertise, and individual patient values and preferences (18, 19).

For OM management, the value of EBN is threefold. First, it grounds nursing interventions in robust scientific evidence—such as data from high-quality randomized controlled trials and systematic reviews—ensuring strategies are scientifically sound and effective (20, 21). Second, clinician expertise contextualizes this evidence, as skilled nurses adapt generalized recommendations to a patient’s specific clinical status, comorbidities, and treatment plan (21, 22). Finally, and pivotally, care plans should incorporate patient values and preferences, such as choices regarding mouthwash use, pain management, or nutritional support (18, 23). This integrated approach is fundamental to developing personalized, effective, and humane OM management strategies that promote patient adherence and improve outcomes.

### 1.3 Aim and framework

This perspective article aims to examine the prevention and management of OM in HSCT patients through an EBN lens. It synthesizes established evidence to propose a structured, actionable, and forward-looking strategic framework. The discussion will first establish the evidence base for OM risk assessment and evaluation. It will then construct a comprehensive management framework integrating primary prevention, secondary intervention, and supportive care throughout the HSCT continuum. Finally, the perspective article will explore the process of translating evidence into practice by addressing implementation barriers and proposing integrative pathways. Ultimately, this perspective study seeks to provide a clear conceptual and practical guide for clinicians and to highlight promising research directions to improve care quality and outcomes.

This perspective article does not seek to reproduce or substitute for established evidence-based guidelines, such as those from the Multinational Association of Supportive Care in Cancer and the European Society for Medical Oncology. Instead, it focuses on the application of guideline evidence within routine nursing practice across the HSCT trajectory. We define a care pathway as a practical framework that details the timing, methodology, and responsible personnel for implementing evidence-based interventions at each clinical stage, thereby extending beyond the general recommendations of clinical guidelines. Consequently, this article addresses the practical gap between evidence and bedside care from a nursing-led implementation perspective.

## 2 Accurate assessment of OM risk and standardization of evaluation criteria

An effective OM prevention and management strategy relies on two key elements: accurately identifying high-risk patients before transplantation and reliably evaluating intervention outcomes afterward. Together, these form the foundation for personalized and precise clinical decision-making.

### 2.1 Application of risk prediction pathways

Precise identification of high-risk patients enables the proactive allocation of resources and targeted preventive strategies (24). While tools like the Oral Mucositis Assessment Scale (OMAS) are valued for objectively quantifying erythema and ulceration, they are of limited pre-transplant value as mucosal damage has not yet occurred (25). An evidence-based approach therefore emphasizes integrating objective tools with patient-specific clinical factors to build a comprehensive predictive pathway (25, 26). Critical risk factors include transplant type (allogeneic carries higher risk than autologous), the mucotoxicity of the conditioning regimen (e.g., containing high-dose melphalan), patient age, and pre-transplant oral health (24, 26). We recommend that this comprehensive risk assessment be adopted as a routine, standardized pre-HSCT procedure. By systematically analyzing these data, clinicians can prioritize patients for intensified prevention, shifting the clinical paradigm from reactive response to proactive prevention.

### 2.2 Standardization of efficacy evaluation criteria

A standardized and reliable method for assessing OM is essential to accurately evaluate intervention outcomes and advance evidence-based practice. Currently, multiple validated grading scales are available, each with distinct characteristics. For example, the WHO scale combines clinical signs with functional impact, such as the patient’s ability to eat (27, 28). The National Cancer Institute’s Common Terminology Criteria for Adverse Events provides detailed, graded symptom descriptions to support clinical documentation and decision-making (27). The OMAS improves sensitivity to changes in severity by quantifying the area of erythema and ulceration at specific oral sites (27–29). However, the variety of tools presents a methodological challenge, hindering direct comparison of results across studies and potentially introducing inconsistency in clinical practice. This variability may compromise continuity of care and weaken the overall evidence base (30, 31). Therefore, we strongly recommend consistently using a single validated assessment instrument within individual institutions and in multicenter research. Such standardization is fundamental to ensuring reliable evaluation, enabling meaningful cross-study comparisons, and supporting the implementation of robust evidence-based care pathways.

## 3 An evidence-based, comprehensive management system

Effective prevention and management of OM in HSCT patients require an evidence-based strategy spanning the entire transplant process. This section outlines a structured management pathway organized around a Prevention-Intervention-Support framework. While oncology nurses have traditionally been central to OM care, their responsibilities have focused primarily on symptom monitoring and supportive care. This pathway expands that role by positioning nurses as key implementers of evidence-based pathways, responsible for conducting dynamic risk assessments, delivering stage-specific interventions, and ensuring continuity of care from pre-transplant preparation through to post-discharge follow-up.

### 3.1 Primary prevention: a proactive approach

Prevention is the cornerstone of OM management, aiming to reduce its severity and delay its onset (32). A fundamental component is standardized basic oral care, which all preventive strategies incorporate (33). Strong evidence supports the implementation of a standardized oral care protocol before transplantation and its continuation throughout the treatment course (34). This protocol should emphasize gentle yet thorough cleansing. Key components include using a soft-bristled toothbrush to minimize trauma and frequent rinsing with non-irritating solutions, such as saline or sodium bicarbonate, to maintain mucosal hydration and clear debris (35, 36). This necessitates collaboration with dentists for pre-transplant oral evaluation and management of any existing dental issues to establish an optimal baseline oral health status. The goal is to preserve the physiological balance of the oral cavity.

For patients receiving specific high-dose chemotherapy (e.g., melphalan) or total body irradiation, palifermin is strongly supported by evidence for significantly reducing the incidence and duration of severe OM (37). It acts as a form of “biological prevention” by stimulating epithelial cell growth and repair. Low-level laser therapy (LLLT) has also demonstrated efficacy in clinical trials for OM prevention, attributed to its anti-inflammatory and tissue-healing properties (38). However, its application is limited by cost, the lack of standardized protocols, and the need for specialized training (39). Cryotherapy (ice chips) is effective for specific agents like 5-fluorouracil or melphalan by cooling the oral mucosa, thereby reducing local blood flow and the delivery of cytotoxic drugs (39, 40). Its use is restricted to the infusion period, and patient tolerance varies (39, 40). Agents such as granulocyte-colony stimulating factor mouthwashes or glutamine supplements are not routinely recommended due to insufficient or inconsistent evidence, despite a theoretical rationale (41).

### 3.2 Secondary prevention and intervention: early recognition and targeted management

When primary prevention is unsuccessful, the focus shifts to early detection and precise intervention to control OM progression and alleviate symptoms. A nurse-led, daily oral assessment using validated tools (e.g., WHO scale or OMAS) is critical for this process (38). Nurses are optimally positioned to detect early signs, such as erythema or patient-reported discomfort, enabling timely intervention before severe ulceration develops (38, 42).

Pain management for OM should be proactive and systematic, typically guided by the principles of the WHO analgesic ladder (43, 44). A key strategy is preemptive analgesia with scheduled dosing rather than “as-needed” administration to prevent the onset of severe pain (45). For mild pain, non-opioids such as acetaminophen may suffice. Moderate to severe pain requires the timely administration of systemic opioids (e.g., morphine, fentanyl), and patient-controlled analgesia can provide effective relief (45, 46). Topical anesthetics (e.g., lidocaine mouthwash) may be used adjunctively before meals to facilitate oral intake, but their use should be limited to avoid side effects such as impaired taste and aspiration risk (38).

In addition to ongoing pain and nutritional management, the therapeutic goals for established ulcers encompass promoting healing and preventing secondary infection. Topical growth factors may accelerate mucosal regeneration (37). Protective barrier films (e.g., sucralfate) can coat ulcers to shield them from further irritation (47). If a secondary fungal or viral infection is suspected, targeted antifungal or antiviral therapy should be initiated, preferably guided by microbiological data.

### 3.3 Supportive care: sustaining vital functions and HR-QOL

Supportive care is essential for maintaining physiological stability and psychological well-being throughout treatment. Nutritional support should adapt as needed, ensuring a transition from an oral diet to enteral and, if required, parenteral nutrition (48, 49). The early involvement of a dietitian is crucial for comprehensive assessment and the development of an individualized plan (49).

Infection control requires careful consideration of risks and benefits. The routine prophylactic use of broad-spectrum antimicrobial or antifungal mouthwashes is not recommended, as it can disrupt the oral microbiome, promote resistance, and lacks strong supporting evidence (38). These agents should be reserved for patients with clear signs of localized infection and, whenever feasible, guided by microbiological identification.

Patients and their families should be engaged as active partners in care (50). Providing structured education on the causes, symptoms, and self-management of OM is fundamental (49). Training in self-management techniques (e.g., pain logging, dietary modifications) enhances patient empowerment and adherence (51). Furthermore, consistent psychological support to address anxiety and depression is vital for improving long-term adherence and HR-QOL.

## 4 From evidence to practice: challenges and integrative pathways

Successfully translating research evidence into routine care is essential for improving patient outcomes, yet this process often encounters significant barriers that require targeted strategies to overcome.

### 4.1 Bridging the evidence-to-practice gap

A persistent gap often separates published guidelines from their application in clinical settings. This disconnect stems from several key factors. First, clinical guidelines can become outdated, failing to incorporate emerging research and leaving clinical nurses at the bedside nurses without current recommendations (52). Second, high workloads and staffing shortages limit the time available for nurses to search for, appraise, and integrate new evidence (53). Third, cost constraints can preclude the adoption of effective but expensive interventions, such as palifermin or LLLT (54). Finally, a lack of awareness of current evidence and deeply ingrained practice habits among staff presents a significant barrier (52). Proactively identifying these challenges is a prerequisite for effective implementation.

Despite these barriers, implementation research confirms that integrating evidence-based OM guidelines into nursing practice yields measurable benefits (45). The adoption of structured oral care protocols has been shown to improve assessment consistency, facilitate earlier symptom recognition, reduce mucositis severity, and enhance patient-reported outcomes (45, 55–57). These findings underscore that the efficacy of guidelines depends fundamentally on their systematic integration into clinical workflows, highlighting the central role of nursing-led strategies—including standardized assessments, structured documentation, and patient education—in translating evidence into tangible patient benefits (57, 58).

For clarity and comparative purposes, Table 1 summarizes key evidence-based OM guidelines and the available evidence concerning their integration into routine clinical practice. This synthesis reveals that while evidence-based guidelines exist, published research on their systematic implementation within standard HSCT nursing care and the assessment of related outcomes remains limited. This evidence gap substantiates the need for the structured, nurse-led clinical care pathway proposed in this perspective article.

**Table 1:** Comparison of major evidence-based guidelines and the proposed nursing-led integrative care pathway for OM in HSCT patients.

Dimension	MASCC/ISOO guidelines	ESMO guidelines	Proposed nursing-led integrative care pathway
Primary purpose	Evidence-based recommendations for OM prevention and treatment	Oncology-focused clinical management recommendations	Translation of evidence into time-sequenced, nurse-led clinical actions specific to HSCT
Target population	Broad oncology population	General cancer population	HSCT patients (autologous and allogeneic)
HSCT specificity	Limited HSCT-specific stratification	HSCT mentioned but not stratified	Explicit differentiation by transplant type, conditioning intensity, neutropenia duration, and GVHD risk
Temporal structure	Not time-based	Not time-based	Phase-oriented: pre-conditioning, conditioning, neutropenia, engraftment, post-discharge
Nursing assessment guidance	General OM assessment recommendation	General OM grading	Detailed nurse-led oral assessment schedule tailored to HSCT timeline
Assessment tools	WHO, NCI-CTCAE, OMAS	WHO, CTCAE	WHO, CTCAE, OMAS, oral mucositis index with institutional standardization
Risk stratification	Limited predictive focus	Limited predictive focus	Integrated risk assessment combining transplant and patient factors
Intervention logic	Evidence strength–based	Symptom severity–based	Risk- and phase-adapted nurse-led decision-making
Implementation strategy	Not addressed	Not addressed	Structured care pathway defining who, when, and how across HSCT phases
Feasibility considerations	Not discussed	Not discussed	Explicit discussion of cost, staffing, training, and real-world constraints
Multidisciplinary integration	Conceptually recommended	Conceptually recommended	Operational nurse-centered coordination across disciplines
Outcome evaluation	Clinical efficacy outcomes	Clinical efficacy outcomes	Clinical, feasibility, and patient-reported outcomes
Evidence-to-practice focus	Evidence synthesis	Evidence synthesis	Implementation-focused translation to bedside nursing practice

MASCC, Multinational Association of Supportive Care in Cancer; ISOO, International Society of Oral Oncology; ESMO, European Society for Medical Oncology; OM, oral mucositis; HSCT, hematopoietic stem cell transplantation; OMAS, Oral Mucositis Assessment Scale. WHO, World Health Organization; NCI-CTCAE, National Cancer Institute’s Common Terminology Criteria for Adverse Events; GVHD, Graft-versus-Host Disease; CTCAE, Common Terminology Criteria for Adverse Events.

### 4.2 Developing structured care pathways

A principal strategy for translating evidence into practice is the adoption of structured care pathways. In this context, a “care pathway” is explicitly distinguished from a clinical guideline. While guidelines provide evidence-based recommendations for what should be done, pathways operationalize these by specifying how, when, and by whom care should be delivered within a specific clinical setting. Consequently, care pathways focus on workflow integration, clear role delineation, and intervention timing—elements fundamental for ensuring consistent implementation in nursing practice.

This approach involves integrating the best available evidence on risk assessment, basic care, pain management, and nutrition into standardized clinical pathways for HSCT patients (59, 60). These pathways provide a timeline, detailing essential assessments and interventions for each transplant phase (60). By establishing a clear protocol, they reduce practice variation, ensure consistent delivery of evidence-based care to all patients, and thereby improve overall care quality (59, 61).

### 4.3 Strengthening the multidisciplinary team framework

Effective OM management requires robust collaboration within a multidisciplinary team (38, 50). In this framework, nurses serve as central coordinators, continuously monitoring patient status, administering interventions, and facilitating communication among all team members. This necessitates close collaboration with physicians on treatment plans, pharmacists on medication management, dietitians on nutritional support, dentists on oral health, and psychologists on addressing patient distress (38, 45). Only through such integrated teamwork can evidence-based guidelines be woven into a seamless, patient-centered care plan, ensuring translation into improved patient outcomes.

## 5 Future perspectives

Although current evidence-based strategies have improved OM management, significant opportunities remain to optimize patient outcomes. Future efforts should focus on addressing critical research gaps and strengthening nursing leadership in this field.

### 5.1 Critical research directions

Several key areas warrant focused research to advance the field. First, effectiveness and implementation research is foundational. Priorities should include pragmatic clinical trials comparing nurse-led OM management pathways to usual care, measuring outcomes such as OM incidence, opioid use, hospital length of stay, and patient-reported HR-QOL (62, 63). Concurrently, implementation science studies are needed to evaluate strategies for integrating evidence-based OM protocols into clinical workflows, for example through electronic health record integration with clinical decision support and automated documentation (64, 65). These studies should systematically assess feasibility and patient-centered outcomes, including nursing workload, protocol adherence, and patient satisfaction, to evaluate a pathway’s real-world effectiveness and sustainability (65, 66). Second, personalized prediction is crucial. Research should investigate biomarkers from genomics, transcriptomics, and the oral microbiome to identify signatures predictive of severe OM, enabling precise risk stratification and preemptive care intensification (67). Third, regarding novel interventions, the potential of mesenchymal stem cell therapy for mucosal regeneration should be explored, alongside the development of advanced biomaterial-based dressings to protect ulcerated surfaces and enhance therapeutic delivery (68). Fourth, digital health technologies hold promise. The development and validation of mobile health applications for remote symptom monitoring and management could improve patient self-efficacy, engagement, and adherence (69). Finally, advancing health economics research is essential (70). Rigorous cost-effectiveness analyses of advanced interventions are required to inform their rational allocation and adoption within healthcare systems (70).

### 5.2 The evolving role of nursing leadership

To meet future challenges in OM management, nurses should proactively expand their leadership roles. While historically serving as clinical nurses at the bedside caregivers, their professional scope is evolving to encompass evidence translation, quality improvement leadership, and digital health integration (71–74). This progression firmly establishes nurses as pivotal agents in bridging the evidence-to-practice gap. Specifically, we advocate for nursing professionals to transcend traditional boundaries by becoming active knowledge translators who systematically disseminate and implement best practices within their institutions (75); clinical change agents who lead initiatives to integrate new evidence and technologies into standardized care pathways (76); and engaged research partners who contribute essential clinical insights to shape research agendas and participate in scientific studies (77). Through demonstrated leadership in these domains, the nursing profession will solidify its indispensable role in advancing OM management and enhancing HR-QOL for HSCT patients (78).

## 6 Summary

In summary, the EBN management of oral mucositis in HSCT patients necessitates a proactive, dynamic, systematic, and individualized strategy. This strategy integrates three essential elements: the application of best available evidence, the implementation of optimized clinical pathways, and effective multidisciplinary collaboration. This integrated approach transforms OM care from a series of isolated interventions into a coordinated, patient-centered continuum. Its consistent application can reduce the clinical burden of OM, alleviate patient suffering, and improve overall outcomes. Ultimately, this represents high-quality nursing care that synthesizes scientific rigor with compassionate practice to enhance the patient experience throughout the HSCT journey.

## Data Availability

The original contributions presented in the study are included in the article/supplementary material, further inquiries can be directed to the corresponding author.
